# Chemical Observations on Functional Nervous Disorders

**Published:** 1867-02

**Authors:** 


*Chemical Observations on Functional Nervous Disorders.* By C. Hanfield Jones, M. B. Cautab; London: F. R. S., Physician to St. Marys' Hospital.

This is an able work. We commend it to the profession.

